# Use of a technology-based system to motivate older adults in performing physical activity: a feasibility study

**DOI:** 10.1186/s12877-021-02021-3

**Published:** 2021-01-28

**Authors:** Els Knippenberg, Annick Timmermans, Steven Palmaers, Annemie Spooren

**Affiliations:** 1 Department of Healthcare, Centre of Expertise – Innovation in Care, PXL University College of Applied Sciences and Arts, Guffenslaan 39, 3500 Hasselt, Belgium; 2 Faculty of Rehabilitation Sciences, REVAL, Hasselt University, Agoralaan Building A, Hasselt University, 3590 Diepenbeek, Belgium; 3 Department of Digital, Centre of Expertise – SMART-ICT, PXL University College of Applied Sciences and Arts, Elfde-Liniestraat 24, 3500 Hasselt, Belgium

**Keywords:** Physical activity, Older adults, Technology, Client-centred

## Abstract

**Background:**

Maintaining or initiating regular physical activity (PA) is important for successful aging. Technology-based systems may support and stimulate older adults to initiate and persevere in performing PA. The aim of the current study was to assess to which extent a customised Kinect system is 1) a credible tool to increase PA in older adults, 2) motivating to perform PA by older adults, and 3) easy to be used in older adults.

**Methods:**

A mixed-method cross-sectional feasibility study was performed in 5 aged care facilities in Flanders, Belgium. Aged participants were asked to perform a 20–30 min test with the intelligent Activity-based Client-centred Training (i-ACT) system. After the test, the ‘Credibility and Expectancy Questionnaire’ (CEQ), the ‘Intrinsic Motivation Inventory’(IMI), the System Usability Scale (SUS), and semi-structured interviews were conducted in the older adults. Feedback was gathered using the thinking aloud method in both aged participants and healthcare professionals.

**Results:**

A total of 48 older adults (20 males and 28 females, mean age = 81.19 (SD = 8.10)), were included. The scores pertaining to system credibility and expectancy, system usability, and motivation towards use were moderate to good. Participants reported that they liked using the i-ACT system, but that the context could be more attractive by adding more visualisations. Twelve professionals stated that they observed involvement in older adults but think that i-ACT is better used in day care centres.

**Conclusions:**

This study indicates that i-ACT is a usable and motivational system to engage older adults to perform PA and therefore supports successful aging. Future research is necessary to investigate the efficacy of i-ACT to perform PA and the transfer to regain and/or maintain engagement in ADLs that older adults find meaningful and purposeful at an older age. Also, further development of i-ACT is advisable to adapt the i-ACT system towards implementation at the home of older adults.

**Trial registration:**

ClinicalTrial.gov ID NCT04489563, 23 July 2020 - Retrospectively registered.

## Background

People worldwide live longer. According to the World Health Organization (WHO) the proportion of the world’s population over 60 years of age will nearly double from 12 to 22% between 2015 and 2050 [1]. The European Commission expects that the old-age dependency ratio (i.e. people aged 65+ relative to aged 15–64) in the European Union will increase from 29% in 2016 to 51% in 2070 [2]. When the years over 60–65 can be lived in good health and in a supportive environment, the ability of older adults to perform their individual meaningful activities adequately will be little different than that of a younger person [1].

Although aging is inevitably related to physical and psychosocial change, this change does not define whether an individual is aging successfully [3]. Successful aging is complex, as it involves not only an individual’s perception, but also professional evaluations (i.e. identify and evaluate different needs in unique individuals to identify the most appropriate intervention) [3]. According to Rowe and Kahn (1997), successful aging is determined by avoiding disease and disability, maintaining high cognitive and physical functioning, and staying involved with life and living [3, 4].

One of the contributing factors of ‘maintaining physical functioning’ is maintaining activity and physical capacity [3, 5, 6]. Physical capacity includes “alleviating impairment and functional limitation”, and “preventing injury, impairment, functional limitation, and disability” [3]. Activity can be described as occupational performance, and refers to “the ability to choose, organize, and satisfactorily perform meaningful occupations that are culturally defined and age appropriate for looking after one’s self, enjoying life, and contributing to the social and economic fabric of a community” [7].

It is not only important to maintain activity and physical capacity when aging [5, 8], but it is also suggested that initiating regular moderate-to-vigorous physical activity, even when older, can improve health and longevity [5, 6]. The WHO defines physical activity as any bodily movement produced by skeletal muscles that requires energy expenditure [9]. However, when aging, people tend to change their behaviour and become more sedentary. According to Fanning et al. (2016), rapid declines in physical function are substantially impacting the overall quality of life of older adults, and also reduce the ability to life independently [5]. Therefore, it is important to stimulate older persons to perform physical activities. According to the WHO, healthy persons of 65 years and above should perform at least 150 min of moderate-intensity aerobic physical activity or at least 75 min of vigorous intensity aerobic physical activity all throughout the week [10]. Although many research is done in older adults with physical exercise programs, with positive results, the most optimal exercise program remains unclear; More detailed research, and specifically in the home environment, is needed [11, 12]. As it is clear that older adults need to perform physical exercises or activities to improve health and longevity, it is also important to stimulate or motivate the person to perform the exercises and activities. To stimulate physical activity (PA) in older adults, centre-based [8] and DVD-based programs [5, 8] at home have been tried and found successful. Especially centre-based PA interventions [8] such as group fitness, group dance, physical games, group gymnastics, etc. However, even if these centre-based PA interventions have effectively improved physical function, they are often costly (e.g. staff costs) and limited in reach (i.e. the amount of persons one can reach) [8, 13].

Most older adults wish to live in their home until they die, i.e. aging in place. To be able to this, older adults need to be able to perform activities of daily life (ADLs). And therefore, they need to maintain strength, balance, and endurance [14]. Not only is it better for the older adults to age in place as safely, independently, and comfortably as possible, it may also provide significant financial advantages regarding health-care expenditures [15].

To stimulate and increase PA in older adults at home, but with a low-cost intervention, technology-based systems are promising tools. Fanning et al. (2016) introduced a DVD, which is a good tool for large-scale intervention delivery, as it is inexpensive, widely known and relatively simple to install and operate. Fanning et al. (2016) suggested that a six month DVD-delivered PA program, was effective for enhancing physical function and quality of life in older adults [5]. Roberts et al. (2017) showed that these positive effects were not maintained at 18 months post-intervention. Although the results of this study were positive, it is shown that there is still a need for PA programs that allow continued participation, especially after the official intervention and the therapist support has ended [8]. Technologies, such as Nintendo Wii, Wii Balance Board or Kinect, are also very popular to stimulate older adults in initiating or maintaining PA [16, 17]. Not only because this technology is also considered low-cost [17, 18], it is also relatively simple to install and operate. Furthermore, although Nintendo Wii or other exergames with computers and/or virtual reality might not always contribute to better outcomes in PA or physical performance [16, 19], it is clear that using exergames provides a positive motivational aspect for PA [16, 18]. To the author’s knowledge, when using Nintendo Wii, Wii Balance Board or Kinect, mostly standard games are used, such as bowling, yoga, ski jump, etc. Only one study was found in which a customised videogame was developed using the Kinect-system [20]. Whether the use of technology motivates older adults s more than other tools to perform physical exercise or activities, is not known to the authors. But motivation itself is an important part in every patient population, hence the importance of motivation in several occupational therapy models and assessments. By using these occupational therapy models, like the Person-Environment-Occupation (PEO-)model or the Canadian Occupational Performance Measure (COPM), therapists can involve the patient more in the process of setting unique and individual goals [7, 21], which increases motivation [22]. This finding is mostly addressed in rehabilitation [22–26], but Vanroy et al. (2019) demonstrated that for older adults to be motivated to exercise, their needs of autonomy, competence and relatedness towards the exercises, need to be satisfied [27]. To fulfil these needs, the use of the aforementioned occupational therapy models and consequently use a client-centred approach, it is assumed by the authors that older adults can be motivated by a technology-based system which incorporates the full client-centred approach to perform PA.

In other physical fields, such as rehabilitation, technology-based system are more common in use and have proven to be effective to motivate persons with disabilities to perform PA. The researchers of this study, developed a technology-based system for use in neurorehabilitation, called the intelligent Activity-based Client-centred Training (i-ACT) system [28]. While it is still a prototype and tested for its effect in functional performance, it showed good results towards usability and motivating people in neurorehabilitation to perform PA, especially because it uses the client-centred approach. As stated above, a client-centred approach is important in every patient population but mostly addressed in rehabilitation [22–25]. Due to these positive results in neurorehabilitation, the researchers would like to gain insights in the use and motivational aspects of the i-ACT in other populations, such as older adults, where physical activity and occupational performance is important.

To explore whether this prototype could also be useful and motivating older adults in performing PA, the aim of this current study was to assess to which extent a customised Kinect system is 1) a credible tool to increase PA in older adults, 2) motivating to perform PA by older adults, and 3) easy to be used by older adults.

## Methods

### Participants and protocol

A cross-sectional feasibility study was performed in five aged care institutes, each was given a period of six weeks, in Flanders, Belgium. A mixed-method approach was used, to combine both quantitative and qualitative data [29]. The choice of also gathering qualitative data was to gain insight in the subjective experience of the users when testing the i-ACT system. The study was approved by the medical ethical committee of Leuven (reference B322201731417). A homogeneous convenience sample [30] of 48 older adults was recruited among elderly in day care centres and nursing homes. Local occupational therapists and other healthcare professionals, such as physiotherapists and patient care assistants, who work with elderly, enrolled eligible persons in the present study based on in- and exclusion criteria.

The older adults had to fulfil the following inclusion criteria: being a client of one of the participating centres, 65 years of age or older, cognitively able to understand and respond to questions, and follow up instructions, understand and speak the Dutch language. Exclusion criteria were: severe spasticity that unable participants to perform exercises, severe visual impairment (e.g. blindness, cataract, etc.), severe communication disorder that unable participants to understand and follow up instructions (e.g. aphasia, agnosia, etc.), persons who use electric wheelchairs who are not able to make a transfer towards a chair.

All aged participants were informed about the study by the head researcher and were included after they gave written informed consent.

Besides older adults, 12 healthcare professionals, participated in the qualitative part of this study. These healthcare professionals were the local therapists, students or care providers of each institute during each period of 6 weeks, and helped and supervised the older adults during the test.

Participants received a 20–30 min test with i-ACT prototype, under supervision of an occupational therapy student and local therapist, concerning different exercises. Exercises consisted of upper limb movements (e.g. reach sideways, upwards, etc.) and/or lower limbs movements (lift right knee, sidestep, light squat, etc.). All exercises were individually determined by the participant’s own therapist in relation to the physical abilities of the participants, based on the professional insight and experience of the therapist and personal interests and ability of the older adult.

After the test, 3 questionnaires were administered by a researcher or occupational student to evaluate the experience of the elderly participants with the i-ACT system. The 3 questionnaires were: the Intrinsic Motivation Inventory (IMI) [31–34], the System Usability Scale (SUS) [35], and the Credibility and Expectancy Questionnaire (CEQ) [36, 37]. None of these questionnaires require a license in order to administer them. The IMI is considered a reliable assessment (intraclass correlation = .70) [34] and was selected to evaluate the motivation to use the i-ACT. It assesses the participant’s subjective experience related to a target activity, in this case the i-ACT exercises. The IMI consists of 6 subscales: interest/enjoyment, perceived competence, effort, felt pressure/tension, value/usefulness, and relatedness. A total IMI-score is not recommended, therefore subscale scores, each with a recalculated maximum score of 7, are used in the analyses [31–34]. The SUS was selected to evaluate the usability of i-ACT within older adults and is also considered a reliable assessment (Cronbach’s alpha = .91) [35]. The item scores on the SUS range from 1 (totally disagree) to 5 (totally agree) and are converted into a score from 0 (negative) to 100 (positive). A score of 72.5 or higher is considered good and above 85.0 is excellent [35]. The CEQ was selected to evaluate the credibility and expectancy with regard to the i-ACT for improvement of PA and is also considered a reliable assessment (Cronbach’s alpha = .85) [37]. The questionnaire consists of 2 subscales, i.e. credibility and expectancy, and scores range from 1 (totally not) to 9 (totally yes) and percentages (0 to 100%). The percentages are converted to scores on a scale of 1 to 9. The maximum score on each subscale is 27. A score of 13.5 is considered neutral, everything above 13.5 is positive while everything under 13.5 is considered negative [36, 37].

During the actual use of the i-ACT for PA exercises, the thinking aloud method was used [38, 39]. This method involves collecting the concurrent verbalisation of a person’s thought while performing a task. As the i-ACT is still under development, it is important to implement as much feedback from the users so the i-ACT is as much fitted towards the wishes and needs of the target group. Therefore, all the thoughts and other feedback that the participants verbalised during the experience with i-ACT were noted. The statements and feedback of the 12 healthcare professionals were also collected using the thinking aloud method during and after the test with i-ACT and noted.

### Apparatus

**Fig. 1:**
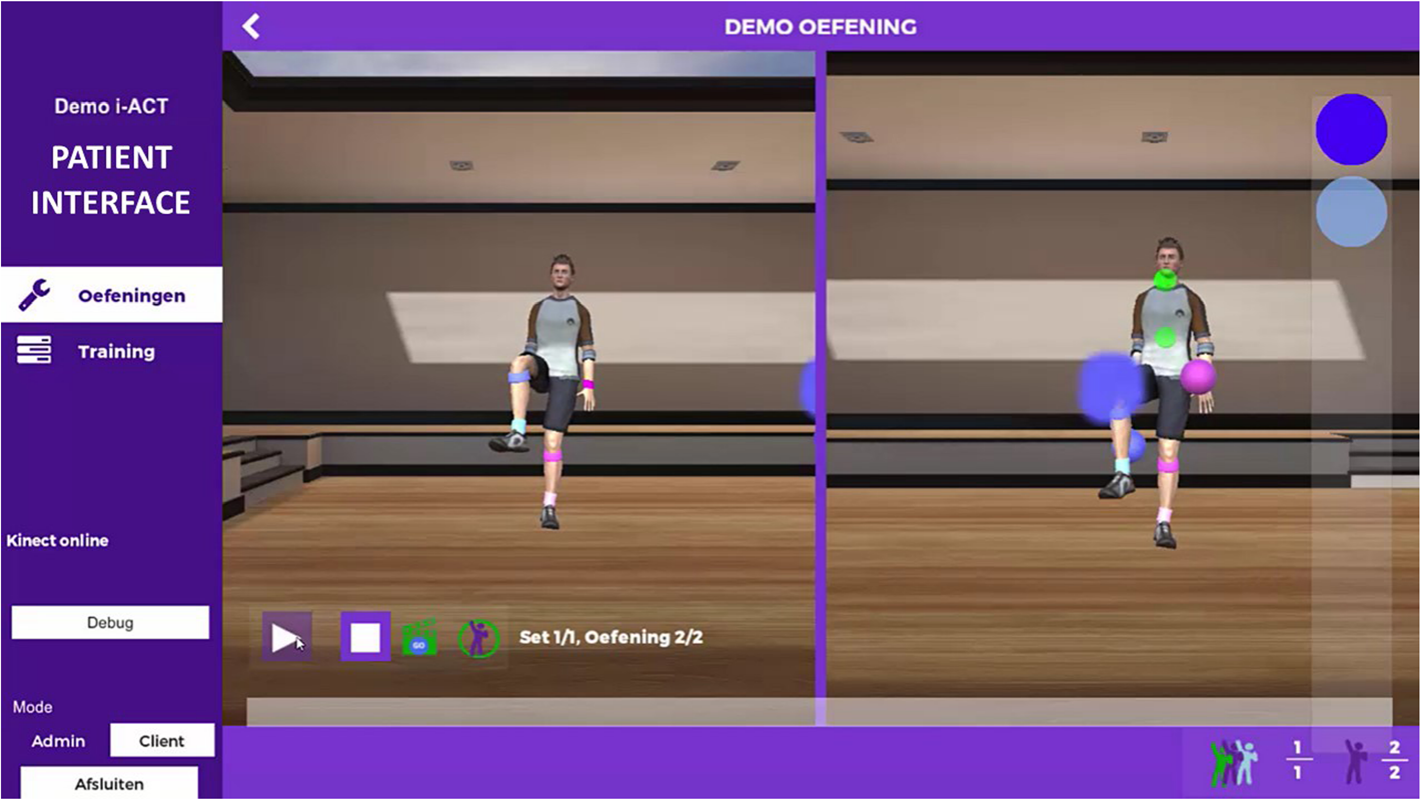
Client interface during performance of the exercise “lifting knee”

The i-ACT prototype was used in this study as a technological system that stimulates physical activity. It is a motion detection system based on a Microsoft Kinect camera and sensors that detect a human shape and human movements. The most important feature of i-ACT is that it is a technology-based system where client-centredness is fully involved in every step. Clinicians record one or more movements which are valuable for the participant, and sets up the necessary parameters to progress towards an exercise (e.g. amount of repetitions, target placement, etc) which is unique for this specific participant [28]. The participant thereby has to follow the example avatar in a virtual environment and has to copy its movement. While performing the movements, the participant receives real time feedback on successful trajectories, stabilisation of body areas during movement (as seen in Fig. 1). After the exercise is finished, feedback is provided on the quality of the participant’s movements.

The details of the software that was used by the i-ACT system are described elsewhere [28].

### Data analyses

Descriptive analysis of the questionnaire results were executed with IBM SPSS Statistics (version 22.0; Armonk, New York).

Qualitative data from the semi-structured interviews in aged participants and the noted statements and feedback gathered using the thinking aloud method in the aged participants and healthcare professionals, were analysed using thematic analysis [40, 41].

## Results

In total, 48 older adults, of which 20 males and 28 females, with a mean age of 81.19 (SD = 8.10), of which 16 persons lived in nursing homes and 32 persons visited day care facilities were included in the feasibility study. Furthermore, 12 healthcare professionals (2 males and 10 females, with a mean age of 29.67 (SD = 8.55), were included in the feasibility study.

### Quantitative assessment

Table 1 includes the descriptive analysis of the 3 outcome measurements, i.e. IMI, SUS and CEQ collected in the aged participants.

**Table 1:** Descriptive data of main outcome measures

Assessment	Scores
**IMI**	
Interest/enjoyment	5.17 (4.61–5.71)
Perceived competence	5.17 (4.95–5.80)
Effort	4.40 (3.80–4.85)
Felt pressure/tension	3.40 (2.55–3.60)
Value/usefulness	5.86 (5.25–6.86)
Relatedness	4.20 (3.40–4.60)
**SUS**	72.50 (67.50–85.00)
**CEQ**	
Credibility	20.50 (16.80–24.00)
Expectancy	15.90 (8.60–19.60)

*IMI* Intrinsic Motivation Inventory (maximum score on all subscales is recalculated to 7);

*SUS* System Usability Scale (maximum score of 100);

*CEQ* Credibility/Expectancy Questionnaire (maximum score on all subscales is 27)

Data of IMI, SUS and CEQ are presented as median (interquartile range)

Regarding the results on the IMI, all but one subscale scored above 4.20/7.00, which indicates good to very good motivation. The median SUS score is 72.50 (67.50–85.00) which indicates a good usability of the system. The CEQ shows a high score on the credibility subscale (= 20.50), while the scores on the expectancy subscale are considered moderately positive (= 15.90).

### Qualitative assessment

**Fig. 2:**
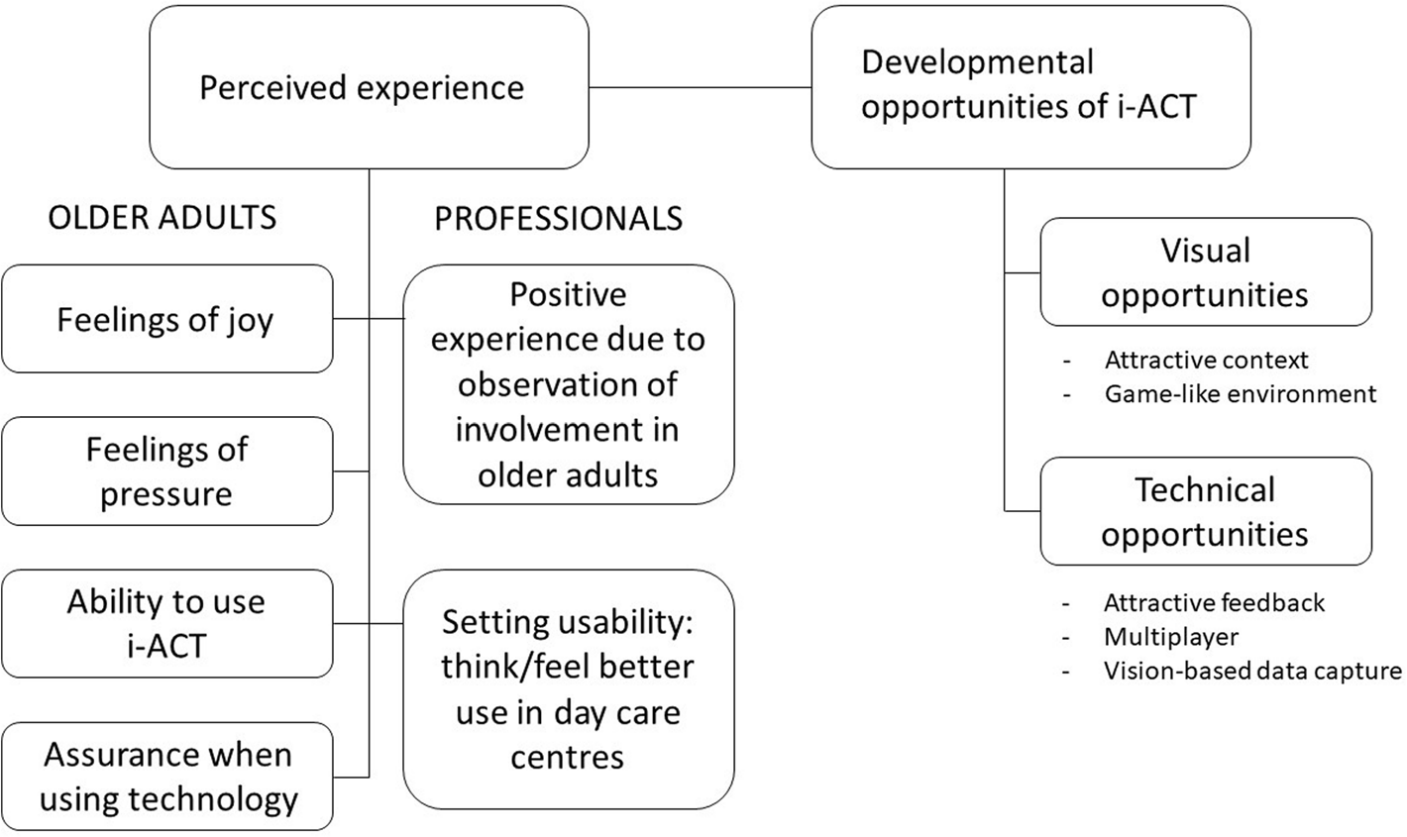
Visual representation of the results of the thematic analysis

Feedback of the users during and after use of the i-ACT system pertained to two main themes, i.e. *perceived experience* and *developmental opportunities* of the i-ACT. Within the perceived experience theme, older adults expressed feelings of joy, pressure, ability, and assurance when using technology. The healthcare professionals mentioned a positive experience with the i-ACT as they observed involvement of the older adults, but also that they think the i-ACT is better used in day care facilities. There were 2 subthemes found in developmental opportunities: visual and technical opportunities. Under visual opportunities, a more attractive context and game-like environment were suggested. Regarding technical opportunities, a more attractive feedback and a multi-player feature were suggested by the users. The fact that i-ACT is based on the Kinect, and thus a vision-based data capture system, was mentioned as a positive developmental opportunity. The themes and subthemes are presented in Fig. 2.

Regarding the first main theme, *perceived experience*, the older adults expressed *feelings of joy* by stating that they liked “*the game”.*

*“The little guy did some weird things that I also had to do. A bit like a mirror, but then with 2 people.”* (Participant 4, female, age-range 75–84, from nursing home facility)*.*

*“I liked it. Maybe not in the beginning because you don’t know what to do. But when you told me to hit those balls, I was on a roll! … It was something new, you know. And you know what, now I can brag about it to my grandkids. Imagine they’re faces as I tell them grandpa used one of those game things”.* (Participant 33, male, age-range 65–74, from day care facility).

There were also older adults who experienced some *feelings of pressure or tension*.

*“I’m not used to working with computers, so I was a bit tensed and anxious to use it beforehand. Luckily the nice girl (i.e. student occupational therapy) comforted me and told me she would stay with me to explain everything.”*

(Participant 27, male, age-range 75–84, from day care facility).

As mentioned in the method section, a prototype was used. So the i-ACT could not be used independently, a healthcare professional as supervisor was necessary. The older adults stated that the presence of the healthcare professional was making them feel more comfortable and better *able to use the i-ACT*.

*“I’m glad you’re here. I could never do this alone.”* After about 10 min of trying exercises, this person stated *“I’m getting the hang of it … thanks to you and your help* (i.e. explanation)*”.* (Participant 41, female, age-range 85+, from day care facility). *“Without X (name of the* healthcare professional*), I would never try things like this. I’m glad I did though. Even at my age … you’re never too old to learn something new.”* (Participant 46, female, age-range 75–84, from day care facility).

Eleven participants clearly stated that they were anxious beforehand about using something new, some sort of technology. But after some time, they felt *assured when using technology*.

“*All that technical stuff, it’s not my cup of tea. I never used a computer or those cordless phones (i.e. mobile phones). So my first thought was, this is not something for me, but when the girl explained to me that it was on the television and showed me what I had to do, I was reassured. I tried it, and I was actually happy that I could do it, you know, make those balloons explode and stuff.”* (Participant 38, female, age-range 85+, from day care facility).

In general the professionals were *positive* about using the i-ACT to stimulate PA in older adults, mostly due to the *involvement* they saw of the participants. The healthcare professionals observed happiness, competition within the participant to reach the targets, etc.

“*It was surprising really, I never saw X (person’s name) engaged so much in an activity, unless during singing or watching an old television program. During other activities we have to keep him involved every 5 minutes or so*”. (Therapist 1, female, age-range 30–40, from nursing home facility).

“*You see that people get more involved the longer they play with it* (i.e. i-ACT*). It’s like they just forget about their surroundings … it’s incredible how focused they are on just a little dot on the screen”*. (Therapist 8, female, age-range 30–40, from day care facility).

Although the healthcare professionals were overall positive about the i-ACT system, taking into account that a prototype was used, they reported that they *think/feel that i-ACT could be better implemented in day care centres* and not so much in nursing homes. The main reason for this is that the healthcare professionals report that people who go to day care facilities have, on average, better cognitive and physical functions than people in nursing homes.

Concerning the second main theme, i.e. *developmental opportunities*, two subthemes were found both in the older adults and healthcare professionals. Regarding the first subtheme, visual opportunities, it can be stated that the participants were overall satisfied with the visual features of the i-ACT system. But six persons would like to have a *more attractive context*, such as a kitchen as background, an open field with clouds and trees, etc. But also a *more “game-like”, interactive environment* such as flying birds that persons need to catch, grasping a cup from a cupboard, kicking against a football, etc.

This is in correspondence with the answers that concern the *technical opportunities*, i.e. the *feedback* “*should not be so plain. You only have those balloons you need to touch and they explode. Can’t you put some birds there and that I have to catch the birds?*” (participant 12, male, age-range 65–74, from nursing home). Furthermore, 17 participants suggested to make “the game” – as called by almost all participants – “a *multiplayer game*, *so that we can play against each other*”.

Another technical opportunity lies in the hard- and software of the Microsoft Kinect. As it uses *vision-based data capture*, no extraneous hardware or sensors on the body are required, and therefore easy to use for older adults as no wires are attached or they do not need to hold a controller.

“*Hey, that guy is doing what I’m doing. How’s that possible? I’m not connected with the tv or anything*”. After explaining that there is a camera in front of him, the person stated, “*how funny. How amazing is technology nowadays. Almost scary! …*. *So the camera sees me, but he doesn’t really see me because I’m not the person on the screen?*” (Participant 37, male, age-range 65–74, from day care facility).

As a prototype of the i-ACT was used, some technical problems occurred and so the feedback of 6 participants was that the system “*should work properly*”. Two people said *“it would be a great thing to get this on my television at home. You know, like those aerobic exercises on television in the old days”.*

## Discussion

The aim of this study was to assess to which extent a customised Kinect system is 1) a credible tool to increase PA in older adults, 2) motivating to perform PA by older adults, and 3) easy to be used in older adults. In general, participants liked using the i-ACT system, as stated by the participants themselves during exercises. The positive perceived experience that the older adults had during and after use of the i-ACT systems, as supported by the quotes, is also visual in the results of the questionnaires. From the results of the CEQ, it can be concluded that the participants have moderate belief that they will improve in PA by exercising with i-ACT, but also that they highly belief that exercising with i-ACT can support them in performing PA. It is assumed that credibility is more connected with the logical thought processes of patients while expectancy is more related to the affective processes [37]. Therefore, from the results of credibility and expectancy towards use of i-ACT, we can assume that older adults are convinced that i-ACT is a system that can help them to perform PA, but that they still need more convincing that i-ACT can improve their physical functioning and thus help them to age successful. This latter can be done by providing older adults with more information [37] about the i-ACT, why PA is important and increase the length of the use of i-ACT. When providing people with more information, more knowledge about the i-ACT, people will increase their use and thus their experience with i-ACT [42]. Furthermore, by providing more information about the i-ACT, older adults might become more at ease with using i-ACT. As seen in the results of the IMI, the results on the subscale “felt pressure/tension”, were the lowest (i.e. 3.40/7.00). While this is low, we can still consider this a good outcome as this score indicates that the older adults felt some level of pressure or tension while performing exercises, but the pressure was not so high that it withheld them from performing the exercises. The same range of results were shown in the pilot study of i-ACT when used in neurorehabilitation. Participants involved in the pilot study and participants of the current study both declared to feel a sort of pressure to perform good on the exercises, i.e. they wanted to hit the targets as best as possible, without compensational movements. Together with the other results on the IMI, it can be suggested that both groups, older adults and persons with central nervous system diseases, are motivated by the i-ACT system to perform good to very good on the exercises provided. Another possible explanation why older adults felt some pressure or tension, might be the fact that older adults have less experience with technology, computers, etc. which can make them anxious, and thus need more information beforehand.

As for the feasibility and usability of i-ACT as a tool for successful aging, we can assume that i-ACT is feasible and usable, based on the results from the mixed-method study. The results of the SUS (mdn 72.50 (67.50–85.00)) suggest that the usability of the i-ACT in older adults is good. These results are supported by the statements of the users, who were generally positive, and were seen in the subthemes “feelings of joy”, “feelings of ability to use the i-ACT” and “positive experience due to observation of involvement in older adults”. According to Chen et al. (2017), who used the Technology Acceptance Model (TAM), perceived usefulness is a valid and reliable predictor of technology use, intentions and attitudes towards working with technology [43]. Furthermore, these results correspond with the findings of the review of Webster and Celik (2014). They found that the Kinect is a promising technology to use in aged care [18]. The first reason is because the Kinect provides the most natural form of human-computer interaction. Secondly, the Kinect is the most feasible technology for a widely dispersed system of elderly exergaming. It uses a vision-based data capture and therefore does not require extraneous hardware [18]. These reasons are in correspondence with the feedback of older adults received during the tests. The last reason mentioned by Webster et al. (2014) is that the Kinect platform is easy to access by developers to create novel and high quality rehabilitation systems and exergames or serious games together with the freedom of controller-free data acquisition [18].

When gathering feedback from the participants in the qualitative part of this study, it appeared that the participants liked using the i-ACT but also suggested some adaptations, such as creating a more attractive context by adding more visualisations of targets or the context of the movement, but still keep it simple. They would have like a more game-like environment such as catching flying birds out of the sky, taking a cup out of the cupboard or even kick against a football. This is in accordance with other systems that work with exergames or serious games in older adults with physical limitations [16, 18–20, 44, 45]. The most research in this area is done in persons with Parkinson’s disease [20, 44, 45]. In these researches it is suggested that persons with Parkinson’s disease are motivated to exercise with virtual reality applications, as well as some positive benefits like increased gait strategies [20, 44]. Also, a multiplayer option should be present. The participants wanted to play against other people, sort of like a competition. It has already been proven that a multiplayer feature motivates people more [46]. Furthermore, in other research it is also suggested to combine a virtual environment with auditory and visual cues to increase exercise intensity and consequently promote fitness in older adults [45]. Although these are good suggestions and they will be considered when developing the i-ACT further with the focus towards older adults, the first focus of i-ACT is still to work as client-centred as possible, as this will also positively influence the motivation of users [46]. Therefore a good combination of working as client-centred as possible, but with more game-like features and implementation of auditory cues, should be taken into account during the following steps in the development of i-ACT for use in aged care.

After the full trial, the healthcare professionals reported that they think/feel that i-ACT could be better implemented in day care centres and not so much in nursing homes. The main reason is that people who go to day care facilities have better cognitive and physical functions than people in nursing homes. Furthermore, the sooner people maintain or initiate moderate to vigorous PA, the more likely they will age successfully, and the better quality of life these people will have when aging [1, 3, 11, 14]. Therefore, there should not only be general guidelines for the general population as generated by the WHO, but also general guidelines for nursing homes and day care facilities regarding the minimum amount of PA training per day or week. Although the benefits of PA in older adults have already been well documented [3, 14], using technology while exercising has the benefits of gathering quantitative data related to physical progress, amount of PA, etc. This is also implemented in i-ACT as not only the time of the execution is stored, but also the amount of targets reached, whether they successfully performed the exercise, and the amount of compensational movements [28]. Although the latter might not be the primary goal in aged care, the other features are also important for future purposes when the system might be used at home in older adults, i.e. caregivers, doctors, or other healthcare professionals can see whether, how much and how well the older adult is performing PA. Furthermore, a Kinect-based system has the advantages of being low-cost, easy to use and controller-free exercises [18, 28, 42, 46]. These advantages are also beneficial when i-ACT is introduced as a PA tool in the home environment of older adults. The physical risks are limited as users are not connected to wires or have to handle a controller. Also, i-ACT is easy to use by the person who performs the exercises, i.e. they have to follow the example avatar in his movements and have to copy them [28].

Methodological considerations also have to be made. This is a feasibility study so only descriptive statistics are performed. Therefore no conclusions can be made to the general aged population. Findings have to be handled with the upmost care and seen in the light of a feasibility study as a first step to perform a higher level of clinical study.

Although the scores were positive on the assessments (i.e. IMI, SUS and CEQ), these scores have to be interpreted with caution as some participants gave socially desirable answers. So the results might be an overestimation of the real thoughts of the participant. Working with persons’ perceptions can have this bias. And although these socially desired answers might be given by the participants, the healthcare professionals and researchers who attended the participant during the exercises, saw involvement of participants, they observed happiness, competition within the participant themselves to reach the targets, etc.

Limitations of this study is also the sample size and the distribution of the sex. The study sample might be enough for a feasibility study, however, a larger sample is necessary and more characteristics of the participants should be gathered, such as education level, level of motor functioning, level of cognitive functioning, experience with technology, etc. Fanning et al. (2016) suggested that people who have at least a college education level, are more likely to provide information and participate in research [5]. Furthermore, the majority of the sample was female and all of them were Caucasian. Although women outnumber men in aged population, they are also more likely to engage in health-related endeavours [5]. Another limitation is that the i-ACT was mostly used in a one-on-one situation and in a separate (part of the) room. Therefore, we cannot formulate the influence of social interaction. In other settings, where the i-ACT is more integrated in the rehabilitation environment, people are getting interested in the i-ACT by seeing it used by peers and interact with the participants and/or healthcare professionals.

The aim of this study was to assess to which extent the i-ACT is a credible, motivational and usable tool to use in older adults to perform PA. The next step would be to investigate the efficacy of the i-ACT to assess whether the i-ACT contributes to increasing the PA of older adults, taking into account the results of this mixed-method feasibility study. For example, a more attractive context or background, implementing some game features so it will become a exergame (e.g. birds as targets), and looking into the possibility of a multiplayer. Regarding outcomes, there should be assessments related to the physical abilities of persons and general health status, and an assessment concerning quality of life and/or life satisfaction. The i-ACT system could be a relatively easy solution to stimulate and maintain PA in older adults in different settings (i.e. home, day care and nursing homes). In relation to the PA protocol, there is no evidence of the most optimal amount of exercises, time and/or intensity. But the guidelines of the WHO are clear and feasible in both care facilities and home environment [10]. The guidelines suggest 1) at least 150 min of moderate-intensity aerobic PA or 75 min of vigorous-intensity aerobic PA throughout the week, 2) aerobic activity in bouts of at least 10 min, and 3) muscle-strengthening activities involving all the major muscle groups at least two days a week [10]. So considering these guidelines, a PA protocol should combine aerobic exercises with muscle-strengthening activities, preferably for at least 30 min per day during at least five days per week, preferably in the morning [27]. The PA program should take into account the general training parameters such as gradual increase in duration, frequency and intensity over time. Also, the program should continue indefinitely, as older adults do not continue their PA program after supervision ends [12, 20]. The PA exercises should be individually set according to the person’s needs and wishes, and by taking into account their preferred occupation/activity in daily life. Even in older adults it is important to define certain goals [26, 47], especially to stimulate PA and focus on regaining and/or maintaining engagement in ADLs that the person finds meaningful and purposeful, even at an older age [3, 7, 48]. This is an advantage of the i-ACT as it is developed as a client-centred tool [28]. The results from this feasibility study are promising as the motivation, usability and credibility are good to very good, so by using a client-centred training program in the next step, it can be expected that the motivation, usability and credibility of the older adults will be even higher. Also it would be important to evaluate the use of a client-centred approach in older adults in different settings, first in day care facilities and nursing homes, and eventually in the home environment.

## Conclusions

The i-ACT is considered to be a credible, usable and motivational tool towards older adults to perform PA, and therefore may contribute to successful aging. Future research should include the efficacy of i-ACT to perform PA in older adults and the transfer to regain and/or maintain engagement in ADLs that they find meaningful and purposeful at an older age. Further development and future research is necessary to adapt the i-ACT system towards implementation at home.

## Data Availability

The datasets used and/or analysed during the current study are available from the corresponding author on reasonable request.

## Abbreviations

ADL: Activities of Daily Life
CEQ: Credibility and Expectancy Questionnaire
COPM: Canadian Occupational Performance Measure
i-ACT: Intelligent Activity-based Client-centred Training
IMI: Intrinsic Motivation Inventory
PA: Physical activity
PEO-model: Person-Environment-Occupation model
SD: Standard deviation
SUS: System Usability Scale
TAM: Technology Acceptance Model
WHO: World Health Organization
